# Detection and classification of SARS‐CoV‐2 using high‐resolution melting analysis

**DOI:** 10.1111/1751-7915.14027

**Published:** 2022-03-01

**Authors:** Liying Sun, Leshan Xiu, Chi Zhang, Yan Xiao, Yamei Li, Lulu Zhang, Lili Ren, Junping Peng

**Affiliations:** ^1^ NHC Key Laboratory of Systems Biology of Pathogens, Institute of Pathogen Biology Chinese Academy of Medical Sciences and Peking Union Medical College Beijing China; ^2^ Key Laboratory of Respiratory Disease Pathogenomics Chinese Academy of Medical Sciences and Peking Union Medical College Beijing China; ^3^ School of Global Health, Chinese Center for Tropical Diseases Research Shanghai Jiao Tong University School of Medicine Shanghai China; ^4^ Christophe Merieux Laboratory, Institute of Pathogen Biology Chinese Academy of Medical Sciences and Peking Union Medical College Beijing China

## Abstract

Coronavirus disease 2019 (COVID‐19), which is caused by severe acute respiratory syndrome coronavirus 2 (SARS*‐*CoV*‐*2), has recently posed a significant threat to global public health. The objective of this study was to develop and evaluate a rapid, expandable and sequencing‐free high‐resolution melting (HRM) approach for the direct detection and classification of SARS‐CoV‐2. Thirty‐one common pathogens that can cause respiratory tract infections were used to evaluate the specificity of the method. Synthetic RNA with serial dilutions was utilized to determine the sensitivity of the method. Finally, the clinical performance of the method was assessed using 290 clinical samples. The one‐step multiplex HRM could accurately identify SARS‐CoV‐2 and differentiate mutations in each marker site within approximately 2 h. For each target, the limit of detection was lower than 10 copies/reaction, and no cross‐reactivity was observed among organisms within the specificity testing panel. The method showed good uniformity for SARS‐CoV‐2 detection with a consistency of 100%. Regarding the clade classification performance, the results showed good concordance compared with sequencing, with the rate of agreement being 95.1% (78/82). The one‐step multiplex HRM method is a rapid method for SARS‐CoV‐2 detection and classification.

## Graphical abstract

The objective of this study was to develop and evaluate a rapid, expandable and sequencing‐free high‐resolution melting (HRM) approach for the direct detection and classification of SARS‐CoV‐2. The one‐step multiplex HRM could accurately identify SARS‐CoV‐2 and differentiate mutations in each marker site. For each target, the limit of detection was lower than 10 copies/reaction, and no cross‐reactivity was observed among organisms within the specificity testing panel.

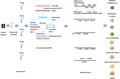

## Introduction

The ongoing pandemic of coronavirus disease 2019 (COVID‐19), which is caused by severe acute respiratory syndrome coronavirus 2 (SARS‐CoV‐2), was initially reported in late December 2019 and now poses an unprecedented threat to global public health and economies. SARS‐CoV‐2 is characterized by an enveloped, single‐stranded positive‐sense RNA (+ssRNA) virus with a genomic length of approximately 30 kb that belongs to the Betacoronavirus genus, which includes two other highly pathogenic coronaviruses that caused recent epidemics: severe acute respiratory syndrome (SARS‐CoV) and Middle East respiratory syndrome (MERS‐CoV), which emerged in 2003 and 2012, respectively (Lu *et al*., 2020; Pachetti *et al*., 2020; Ren *et al*., 2020; Xie *et al*., 2020; Yan *et al*., 2020). Although SARS‐CoV and MERS‐CoV infections have higher mortality rates than SARS‐CoV‐2, SARS‐CoV‐2 spreads much more rapidly than MERS‐CoV and SARS‐CoV (Ogando *et al*., 2020; Petersen *et al*., 2020; To *et al*., 2020). As of July 8th, 2021, more than 200 countries have been affected by the virus, and despite all intervention and control measures, the number of laboratory‐confirmed COVID‐19 confirmed cases has risen above 184 million with over 3.9 million deaths (https://covid19.who.int/). Currently, there were more than 100 candidate vaccines under development according to the World Health Organization (WHO). However, it may take some time before a broader application of the COVID‐19 vaccine becomes viable. Multiplex studies have confirmed that the ability to quickly detect COVID‐19 infection as early as possible (optimally at the earliest appearance of symptoms) is crucial for controlling the spread of disease.

Generally, the mutation rate of RNA viruses is dramatically high because of the lack of intrinsic proofreading and repair capability for correcting replication errors; thus, they have been evolving continuously with new mutations for viral adaptation, which inevitably face natural selective pressures imposed by the host (Pachetti *et al*., 2020; Xing *et al*., 2020). Despite the sluggish mutation rate of the virus in the current pandemic, potential genetic mutations of SARS‐CoV‐2 that make it highly pathogenic and difficult for specific drug or preventive vaccine development have raised concerns about curbing the pandemic in the indefinite future. The amino acid substitution D614G (SNP mutation 23403A > G) outside the receptor‐binding domain of SARS‐CoV‐2 is rapidly becoming dominant over time in different regions, and several recent studies have suggested that it yields a more stable phenotype with a significant fitness advantage and higher transmission efficacy (Korber *et al*., 2020; Naqvi *et al*., 2020; Pachetti *et al*., 2020; Yin, 2020). Considering the role of sequence data as one of the most important aspects for providing invaluable insights into the ongoing evolution and epidemiology of the virus during the pandemic, current research is focused on the correlation between transmission dynamics and the genotype of SARS‐CoV‐2 (Rambaut *et al*., 2020). Efficient viral subtyping enables visualization and modelling of the geographic distribution and temporal dynamics of disease spread (Zhao *et al*., 2020). Implementing real‐time genomic surveillance could further enhance our understanding of how to develop effective containment strategies and design new vaccines, antiviral drugs and diagnostic assays (Alm *et al*., 2020; Pachetti *et al*., 2020). Based on the genetic relatedness of the sequences, SARS‐CoV‐2 has been divided into eight high‐level phylogenetic clades using significant marker mutations: S, L, V, G, GH, GR, GV and GRY (https://www.gisaid.org/references/statements‐clarifications/clade‐and‐lineage‐nomenclature‐aids‐in‐genomic‐epidemiology‐of‐active‐hcov‐19‐viruses/). Systematically tracking major clades of SARS‐CoV‐2 is therefore important, as it allows monitoring of the molecular epidemiology of circulating viral sequences nationally and internationally (Alm *et al*., 2020; Guan *et al*., 2020; Korber *et al*., 2020; Mercatelli and Giorgi, 2020). In order to enable a quick response to a potential outbreak, it is desirable to have a fast, extensible, accurate and comprehensive diagnostic method capable of simultaneously detecting significant mutations and subtyping SARS‐CoV‐2.

In this study, we propose a methodology based on high‐resolution melting (HRM) analysis for SARS‐CoV‐2 detection that can rapidly and consistently identify the virus, subtypes and mutations without requiring a sequencing analysis step, thereby avoiding the need for reagents and/or supplies. HRM is a convenient, extensible, reliable, closed‐tube and cost‐effective approach that has been used for identification of species or mutation scanning or molecular typing in several research fields, such as epidemiology and microbiology (Montgomery *et al*., 2010; Tong and Giffard, 2012; Li *et al*., 2014; Xiu *et al*., 2020b; Xiu *et al*., 2020). By exploring the advantages of HRM analysis, we developed a multiplex assay using reverse transcription coupled with HRM analysis of the amplification products to rapidly detect viral RNA and simultaneously identify mutations in SARS‐CoV‐2. This development opens the door to the rapid initiation of effective strategies to curb COVID‐19 outbreaks.

## Results

### Developing and optimizing the one‐step multiplex HRM assay

**Fig. 1:**
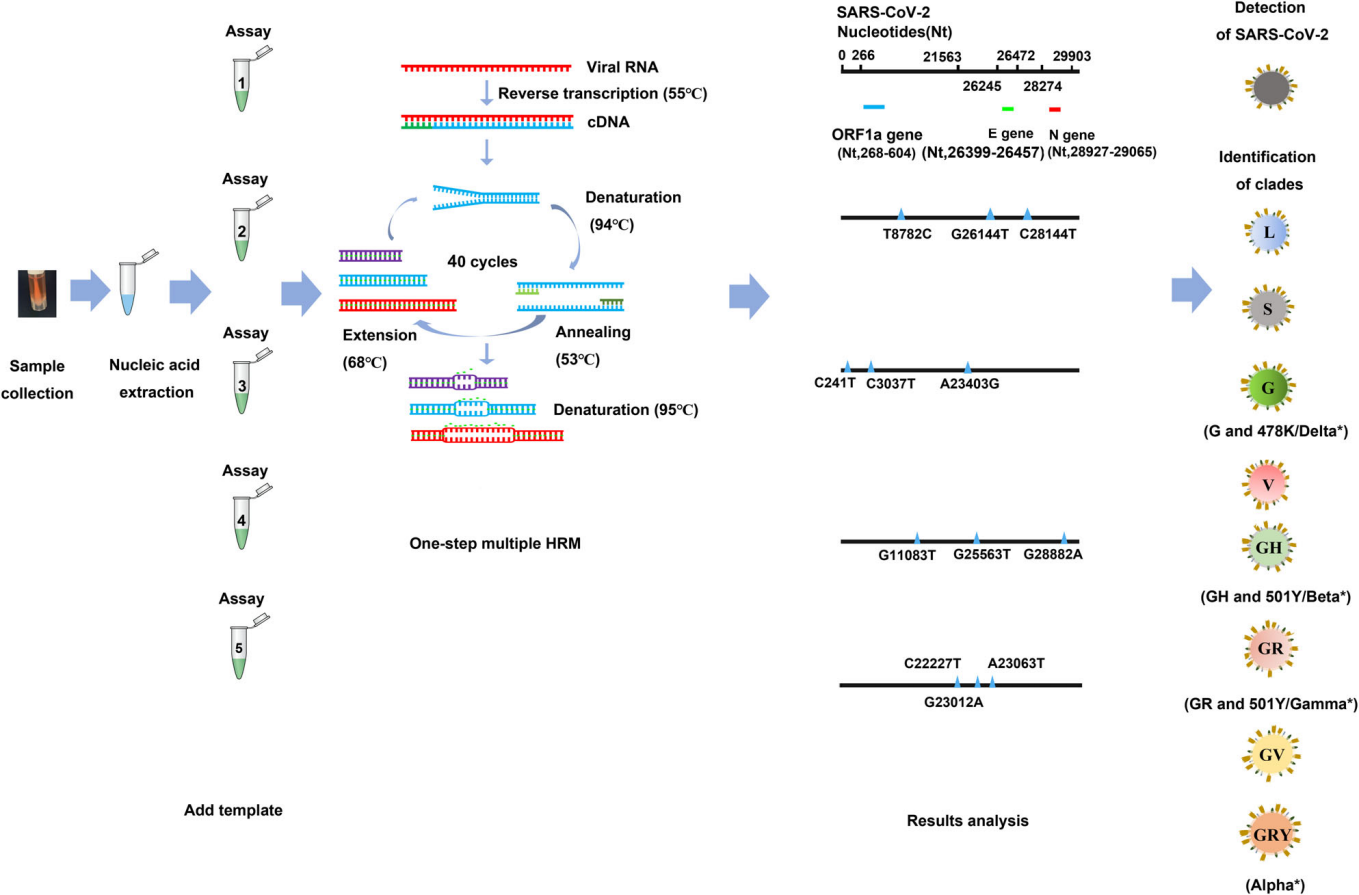
The experimental workflow of the one‐step multiplex HRM method. The workflow contained five steps. First, respiratory samples were collected and underwent nucleic acid extraction. Then, RNA was added to five assays: quadruplex assay 1 was used for identifying SARS‐CoV‐2, and four triplex assays (assay 2, 3, 4 and 5) were used for clade classification. Samples were regarded as SARS‐CoV‐2‐positive only if the specific melting peaks of all three targets in assay 1 (the ORF1a, nucleocapsid and envelope genes) were observed. Marker mutations were distinguished according to the corresponding *T*_m_ value inferred from the melting curves. Finally, clades were identified based on the profiles of twelve marker mutations using the GISAID nomenclature. *SARS‐CoV‐2 variants are named according to World Health Organization (WHO) assigned labels.

**Fig. 2:**
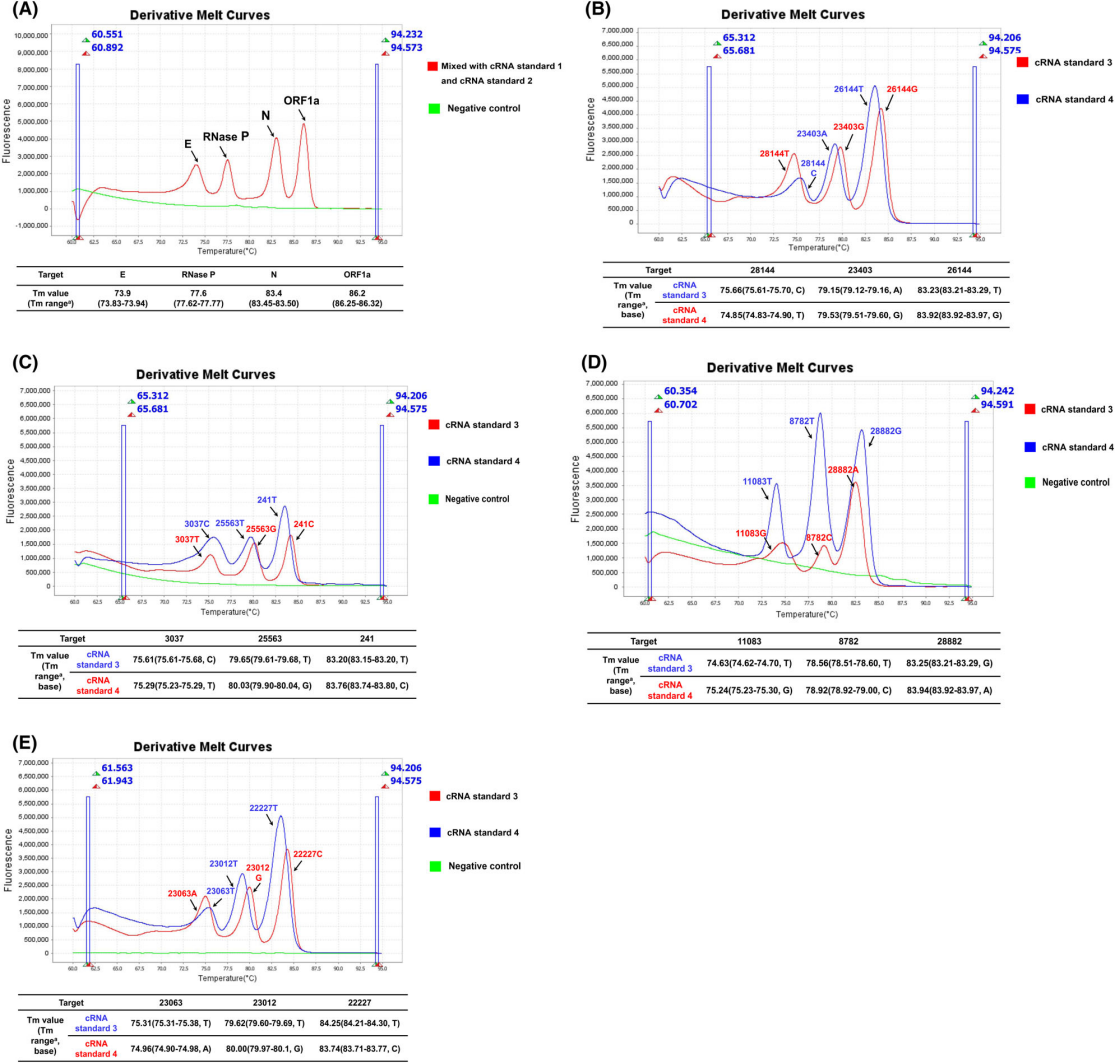
Melting curves of the optimized assays used for the detection of complementary RNA (cRNA) standards. (A) cRNA standards 1, 2 and the negative control (nuclease‐free water) were tested using assay 1, and the results showed four separated peaks representing four targets (ORF1a, nucleocapsid, envelope and human Rnase P). (B–D) cRNA standards 3, 4 and nuclease‐free water were tested using assays 3, 4 and 5 respectively, and the results showed that different mutations in each marker site could be clearly discriminated. The ‘a’ means the *T*_m_ value range of results interpretation criteria in Tables 2 and 3.

In this study, we developed and assessed a one‐step multiplex HRM method for the detection of SARS‐CoV‐2 and the rapid classification of clades, which consists of one quadruplex assay (SARS‐CoV‐detecting assay, assay 1) and four triplex assays (SARS‐CoV‐2 clustering assays, assay 2–5). To increase the specificity of SARS‐CoV‐2 detection, the quadruple detecting assay targets three different regions (ORF1a, N and E) of the viral genome and targets the human RNase P gene as an internal control to ensure the success of sampling and nucleic acid extraction. To provide more detailed phylogenetic information, four triple clustering assays were used for analysing twelve marker mutations within eight high‐level phylogenetic groupings, according to the latest nomenclature system for major clades introduced by GISAID. The twelve marker mutations are as follows: 241C/T, 3037C/T, 8782C/T, 11083G/T, 23403A/G, 25563G/T, 26144G/T, 28144T/C, 28882G/A, 23063A/T, 23012G/A, 22227C/T. The primer locations and experimental workflow are illustrated in Fig. 1. As shown in Fig. 2, the synthetic RNA standards (Standard 1, 2, 3 and 4) were tested by specific combinations for one‐step multiplex HRM method. Specifically, standard 1, 2, 3 and 4 were used as a standard for the analysis of the viral genome (ORF1a, N and E), internal control (human RNase P) and mutation sits (standards 3 and 4) respectively (Table S2). The peaks of amplicons could be clearly observed in Fig. 2, and for clustering assays, each variant could be differentiated clearly via the discrete *T*_m_ values (Melting temperature values, Fig. 2B–E). Moreover, the optimized concentrations of primers allowed consistent height of the curve peaks. Final primer sequences, volumes added in each assay, and target genes are listed in Table 1. The *T*_m_ value range of amplicons in detecting assay (ORF1a, N, E and RNase P) and four clustering assays are shown in Tables 2 and 3, as the results interpretation criteria for the one‐step multiplex HRM methods. According to the *T*_m_ value measured by one‐step multiplex HRM method and the results analysis standard (Tables 2 and 3), whether a sample was SARS‐CoV‐2‐positive and the base of marker sites were determined, and then the clade was identified.

**Table 1:** Details of the five assays used in the one‐step HRM method.

Assay	Target	Primer	Sequence (5’‐3’)^a^	Volume (μl)^b^	Mutation
Assay 1	ORF1a	ORF1a‐F	GGAGAGCCTTGTCCCTGGTTTCAACGAG	0.5	–
		ORF1a‐R	TTCGCCCACATGAGGGACAAGGACA	0.5	
	N	N‐F	TCTTGCTTTGCTGCTGCTTGACAGA	0.5	–
		N‐R	GCAGTACGTTTTTGCCGAGGCTT	0.5	
	E	E‐F	TAAAACCTTCTTTTTACGTTTACTCTCG	0.5	–
		E‐R	GGAACTCTAGAAGAATTCAGATTTTTAAC	0.5	
	RNase P	Rnase P‐F	GCAAAGCATCGGACTGAACC	1	‐
		Rnase P‐R	ACCCGCAGAACAGTTGTCTT	1	
Assay 2	ORF8	28144‐F	GTTCTAAATCACCCATTCAGTACAT	0.5	L84S
		28144‐R	CCAATTTAGGTTCCTGGCAATTA	0.5	
	S	23403‐F	CACCAGGAACAAATACTTCTAACCAGG	0.5	D614G
		23403‐R	GTAGAATAAACACGCCAAGTAGGAGTAAG	0.5	
	ORF3a	26144‐F	GCGCGCGTTGATGAGCCTGAAGAACATGTC	0.5	G251V
		26144‐R	GCGCGCGCTTGTGCTTACAAAGGCACG	0.5	
Assay 3	ORF1ab	3037‐F	CTGGTGAGTTTAAATTGGCTTCACATATG	1	Synonymous mutation
		3037‐R	TCAAACTCTTCTTCTTCACAATCACCTTC	1	
	ORF3a	25563‐F	GCTTATTGTTGGCGTTGCACTTCT	0.5	Q57H
		25563‐R	CTTGGAGAGTGCTAGTTGCCATCTC	0.5	
	5’ UTR	241‐F	TCGTCCGTGTTGCAGCC	0.5	Synonymous mutation
		241‐R	CCAGGGACAAGGCTCTCCA	0.5	
Assay 4	ORF1ab	11083‐F	GCGCAGAGTACTCAATGGTCTTTGTTCTTTTTTT	1	L3606F
		11083‐R	GCGCATAGCAAAAGGTAAAAAGGCATTTTCAT	1	
	ORF1a	8782‐F	ACTCGTGACATAGCATCTACAG	0.5	Synonymous mutation
		8782‐R	TGCAGCAATCAATGGGCAA	0.5	
	N	28882‐F	GGCGGCAGTCAAGCC	1	R203K
		28882‐R	CCGCCATTGCCAGCC	1	
Assay 5	S	23063‐F	CCTTTACAATCATATGGTTTCCAACCCAC	1	N501Y
		23063‐R	CTCTGTATGGTTGGTAACCAACACCA	1	
	S	23012‐F	GCCGCGGTAGCACACCTTGTAATGGTG	1	E484K
		23012‐R	TGGGTTGGAAACCATATGATTGTAAAGGAAAG	1	
	S	22227‐F	GCGCGCGCGCGCAGTGCGTGATCTCCCTCAG	1	A222V
		22227‐R	CCTATTGGCAAATCTACCAATGGTTCT	1	

^a^ Underlined bases represent the G or GC tails added to the 5’ end of some primers.

^b^ Volume of each 10 μM primer added to the primer pools.

**Table 2:** The results interpretation criteria of the one‐step multiplex HRM methods (Assay 1 and 2).

Assay		1				2		
Mutation sites		E	RNase P	N	ORF1a	28144	23403	26144
		*T*_m_ value range (95% CI) base						
Clades^b^	L^a^	73.83–73.94	77.62–77.77	83.45–83.50	86.25–86.32	(74.83–74.90) **T**	(79.12–79.16) **A**	(83.92–83.97) **G**
	S	73.83–73.94	77.62–77.77	83.45–83.50	86.25–86.32	(75.61–75.70) **C**	(79.12–79.16) **A**	(83.92–83.97) **G**
	V	73.83–73.94	77.62–77.77	83.45–83.50	86.25–86.32	(74.83–74.90) **T**	(79.12–79.16) **A**	(83.21–83.29) **T**
	G	73.83–73.94	77.62–77.77	83.45–83.50	86.25–86.32	(74.83–74.90) **T**	(79.51–79.60) **G**	(83.92–83.97) **G**
	GH	73.83–73.94	77.62–77.77	83.45–83.50	86.25–86.32	(74.83–74.90) **T**	(79.51–79.60) **G**	(83.92–83.97) **G**
	GR	73.83–73.94	77.62–77.77	83.45–83.50	86.25–86.32	(74.83–74.90) **T**	(79.51–79.60) **G**	(83.92–83.97) **G**
	GV	73.83–73.94	77.62–77.77	83.45–83.50	86.25–86.32	(74.83–74.90) **T**	(79.51–79.60) **G**	(83.92–83.97) **G**
	GRY	73.83–73.94	77.62–77.77	83.45–83.50	86.25–86.32	(74.83–74.90) **T**	(79.51–79.60) **G**	(83.92–83.97) **G**

CI, confidence interval.

^a^ The SARS‐CoV‐2 reference strain (GenBank accession no. NC_045512.2).

^b^ The underlined base means the mutation site.

**Table 3:** The results interpretation criteria of the one‐step multiplex HRM methods (Assay 3, 4, and 5).

Assay		3			4			5		
Mutation sites		3037	25563	241	11083	8782	28882	23063	23012^b^	22227
		*T*_m_ value range (95% CI) base								
Clades^c^	L^a^	(75.61–75.68) **C**	(79.90–80.04) **G**	(83.74–83.80) **C**	(75.23–75.30) **G**	(78.92–79.00) **C**	(83.92–83.97) **G**	(74.90–74.98) **A**	(79.97–80.1) **G**	(83.71–83.77) **C**
	S	(75.61–75.68) **C**	(79.90–80.04) **G**	(83.74–83.80) **C**	(75.23–75.30) **G**	(78.51–78.60) **T**	(83.92–83.97) **G**	(74.90–74.98) **A**	(79.97–80.1) **G**	(83.71–83.77), **C**
	V	(75.61–75.68) **C**	(79.90–80.04) **G**	(83.74–83.80) **C**	(74.62–74.70) **T**	(78.92–79.00) **C**	(83.92–83.97) **G**	(74.90–74.98) **A**	(79.97–80.1) **G**	(83.71–83.77) **C**
	G	(75.23–75.29) **T**	(79.90–80.04) **G**	(83.15–83.20) **T**	(75.23–75.30) **G**	(78.92–79.00) **C**	(83.92–83.97) **G**	(74.90–74.98) **A**	(79.97–80.1) **G**	(83.71–83.77) **C**
	GH	(75.23–75.29) **T**	(79.61–79.68) **T**	(83.15–83.20) **T**	(75.23–75.30) **G**	(78.92–79.00) **C**	(83.21–83.29) **A**	(74.90–74.98) **A**	(79.97–80.1) **G**	(83.71–83.77) **C**
	GR	(75.23–75.29) **T**	(79.90–80.04) **G**	(83.15–83.20) **T**	(75.23–75.30) **G**	(78.92–79.00) **C**	(83.92–83.97) **G**	(74.90–74.98) **A**	(79.97–80.1) **G**	(84.21‐84.30) **T**
	GV	(75.23–75.29) **T**	(79.90–80.04) **G**	(83.15–83.20) **T**	(75.23–75.30) **G**	(78.92–79.00) **C**	(83.21–83.29) **A**	(75.31–75.38) **T**	(79.97–80.1) **G**	(83.71–83.77) **C**
	GRY	(75.23–75.29) **T**	(79.90–80.04) **G**	(83.15–83.20) **T**	(75.23–75.30) **G**	(78.92–79.00) **C**	(83.92–83.97) **G**	(74.90–74.98) **A**	(79.60–79.69) **T**	(83.71–83.77) **C**

CI, confidence interval.

^a^ The SARS‐CoV‐2 reference strain (GenBank accession no. NC_045512.2).

^b^ Mutation sites 23012 plays an important role in SARS‐CoV‐2 infection and immune escape (was not used for clustering).

^c^ The underlined base means the mutation site.

### Analytical performance of the method

Each dilution of cRNA standards 1, 2, 3 and 4 was repeatedly tested 11 times, and the LOD (Limit of detection) was determined using probit regression analysis at the 95% detection level. For each target, the LOD was lower than 10 copies/reaction (Table 4). All of the samples in the specificity testing panel were detected negative using one‐step multiplex HRM assay 1, and there was also no cross‐reaction between targets within assays 2–5 and the specificity testing panel (Table 5).

**Table 4:** The LOD of each target calculated using regression probit analysis.

Targets	No. of positive/No. of replicates (%) for each dilution copies/reactions								LOD copies/reaction (95% CI)
	10 000	1000	100	50	20	10	5	1	
ORF1a	11/11 (100)	11/11 (100)	11/11 (100)	11/11 (100)	11/11 (100)	11/11 (100)	9/11 (81.8)	6/11 (54.5)	7.38 (4.25–35.16)
N	11/11 (100)	11/11 (100)	11/11 (100)	11/11 (100)	11/11 (100)	11/11 (100)	9/11 (81.8)	6/11 (54.5)	7.38 (4.73–35.16)
E	11/11 (100)	11/11 (100)	11/11 (100)	11/11 (100)	11/11 (100)	11/11 (100)	7/11 (63.6)	4/11 (36.4)	8.90 (6.30–20.91)
RNase P	11/11 (100)	11/11 (100)	11/11 (100)	11/11 (100)	11/11 (100)	11/11 (100)	8/11 (72.7)	4/11 (36.4)	8.06 (5.62–19.73)
8782	11/11 (100)	11/11 (100)	11/11 (100)	11/11 (100)	11/11 (100)	11/11 (100)	6/11 (54.5)	4/11 (36.4)	9.72 (6.90–22.12)
28144	11/11 (100)	11/11 (100)	11/11 (100)	11/11 (100)	11/11 (100)	11/11 (100)	5/11 (45.5)	3/11 (27.3)	9.96 (7.31–19.54)
26144	11/11 (100)	11/11 (100)	11/11 (100)	11/11 (100)	11/11 (100)	11/11 (100)	5/11 (45.5)	3/11 (27.3)	9.96 (7.31–19.54)
241	11/11 (100)	11/11 (100)	11/11 (100)	11/11 (100)	11/11 (100)	11/11 (100)	10/11 (90.9)	5/11 (45.5)	5.80 (3.80–21.54)
3037	11/11 (100)	11/11 (100)	11/11 (100)	11/11 (100)	11/11 (100)	11/11 (100)	8/11 (72.7)	3/11 (27.3)	7.80 (5.58–16.95)
23403	11/11 (100)	11/11 (100)	11/11 (100)	11/11 (100)	11/11 (100)	11/11 (100)	10/11 (90.9)	5/11 (45.5)	5.80 (3.80–21.54)
28882	11/11 (100)	11/11 (100)	11/11 (100)	11/11 (100)	11/11 (100)	11/11 (100)	10/11 (90.9)	7/11 (63.6)	8.25(4.91–34.98)
25563	11/11 (100)	11/11 (100)	11/11 (100)	11/11 (100)	11/11 (100)	11/11 (100)	10/11 (90.9)	7/11 (63.6)	8.25 (4.91–34.98)
11083	11/11 (100)	11/11 (100)	11/11 (100)	11/11 (100)	11/11 (100)	11/11 (100)	5/11 (45.5)	3/11 (27.3)	9.96 (7.31–19.54)
23063	11/11 (100)	11/11 (100)	11/11 (100)	11/11 (100)	11/11 (100)	11/11 (100)	5/11 (45.5)	2/11(18.2)	9.51 (7.16–17.1)
23012	11/11 (100)	11/11 (100)	11/11 (100)	11/11 (100)	11/11 (100)	11/11 (100)	6/11 (54.5)	3/11(27.3)	9.33 (6.80–18.72)
22227	11/11 (100)	11/11 (100)	11/11 (100)	11/11 (100)	11/11 (100)	11/11 (100)	6/11 (54.5)	2/11(18.2)	8.92 (6.67–16.52)

CI, confidence interval; LOD, limit of detection.

**Table 5:** The specificity evaluation of one‐step multiplex HRM method.

Number	Sample	No.^c^	Assay 1^c^	Assay 2^d^	Assay 3^e^	Assay 4^f^	Assay 5^g^
1	SARS‐CoV‐2 virus	4	+	+	+	+	+
2	Adenovirus	1	−	−	−	−	−
3	Human enterovirus	1	−	−	−	−	−
4	Human coronaviruses OC43	1	−	−	−	−	−
5	Human coronaviruses 229E	1	−	−	−	−	−
6	Human coronaviruses NL63	1	−	−	−	−	−
7	Human coronaviruses HKU1	1	−	−	−	−	−
8	Middle East respiratory syndrome CoV	1	−	−	−	−	−
9	Human bocavirus 1	1	−	−	−	−	−
10	Human metapneumoviruses A	1	−	−	−	−	−
11	Human metapneumoviruses B	1	−	−	−	−	−
12	Human rhinovirus	1	−	−	−	−	−
13	Influenza A H1N1	1	−	−	−	−	−
14	Influenza A H3N2	1	−	−	−	−	−
15	Influenza B viruses	1	−	−	−	−	−
16	Parainfluenza virus 1	1	−	−	−	−	−
17	Parainfluenza virus 2	1	−	−	−	−	−
18	Parainfluenza virus 3	1	−	−	−	−	−
19	Parainfluenza virus 4	1	−	−	−	−	−
20	Respiratory syncytial viruses A	1	−	−	−	−	−
21	Respiratory syncytial viruses B	1	−	−	−	−	−
22	SARS‐Like coronavirus^b^	1	−	−	−	−	−
23	*Legionella pneumophila*	1	−	−	−	−	−
24	*Bordetella pertussis*	1	−	−	−	−	−
25	*Mycoplasma pneumoniae*	1	−	−	−	−	−
26	*Chlamydophila pneumoniae*	1	−	−	−	−	−
27	*Haemophilus influenzae*	1	−	−	−	−	−
28	*Staphylococcus aureus*	1	−	−	−	−	−
29	*Moraxella catarrhalis*	1	−	−	−	−	−
30	*Klebsiella pneumoniae*	1	−	−	−	−	−
31	*Pseudomonas aeruginosa*	1	−	−	−	−	−
32	*Acinetobacter baumannii*	1	−	−	−	−	−
33	*Streptococcus pneumoniae*	1	−	−	−	−	−
34	*Escherichia coli*	1	−	−	−	−	−
35	*Neisseria meningitidis*	1	−	−	−	−	−

+, positive detection; ‐, negative detection.

^a^ This sample was collected from bat.

^b^ The number of samples.

^c^ The detecting assay of one‐step multiplex HRM method.

^d,e,f and g^ The clustering assays of one‐step multiplex HRM method.

### Applying one‐step multiplex HRM to clinical samples

RNA from 280 nasopharyngeal swabs and 10 sputum samples collected from patients with pneumonia or suspected SARS‐CoV‐2 infection were simultaneously detected using qRT‐PCR and one‐step multiplex HRM. For one‐step multiplex HRM, samples were regarded as SARS‐CoV‐2 positive only if specific melting peaks of all three targets in assay 1 (the ORF1a, N and E genes) were observed. If not, testing was repeated for further confirmation. According to the criteria, 82 out of 290 samples were tested positive using one‐step multiplex HRM method, and were in concordance with the results of qRT‐PCR. The remaining 208 samples were tested as SARS‐CoV‐2 negative by both of the two methods. As to the detecting assay, the one‐step multiplex HRM method showed good consistency with qRT‐PCR.

Then, according to the results of clustering assays in the one‐step HRM method and interpretation criteria (Tables 2 and 3), the bases of twelve marker mutations of 82 SARS‐CoV‐2‐positive samples were determined and were then compared with the results of Sanger sequencing or ARTIC amplicon sequencing, in order to verify the accuracy of clustering assays. Except four samples with qRT‐PCR *C*_t_ values > 35, the bases of marker mutations in other 78 samples were identical with those determined by sequencing, with the rate of agreement being 95.1% (78/82). The results demonstrated that although the interpretation criteria was defined based on the *T*_m_ values obtained from synthetic RNA, it can completely fit the situation of analysing RNA extracted from clinical specimens.

**Fig. 3:**
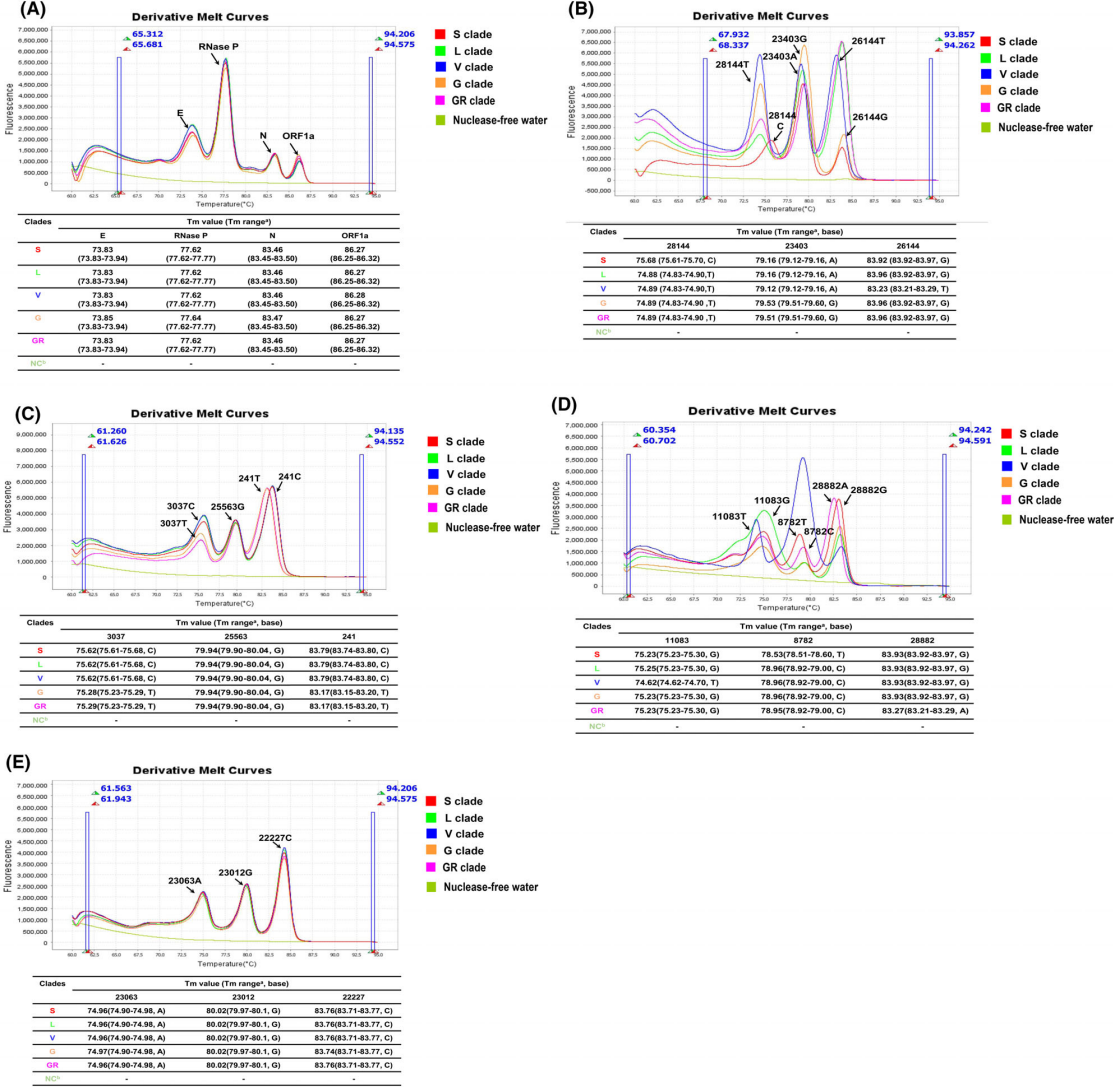
Melting curve of the one‐step multiplex HRM method used for the identification of clinical strains and samples. (A) Clinical specimens of clades S, L, V, G and GR, and the negative control (nuclease‐free water) were analysed using assay 1. The results showed four independent peaks representative of four targets (ORF1a, nucleocapsid, envelope and human Rnase P genes). (B–E) Clinical specimens of clades S, L, V, G and GR, and the negative control (nuclease‐free water) were detected using assays 2, 3, 4 and 5 respectively. The results indicated that twelve mutations could be clearly distinguished and were consistent with the sequencing results. ‘a’ means the *T*_m_ value range of results interpretation criteria in Tables 2 and 3. ‘b’ was the negative control (nuclease‐free water).

Finally, profiles of the twelve marker mutations of 82 SARS‐CoV‐2‐positive samples were classified into clades accordingly. Classification was based on the latest GISAID nomenclature (https://www.gisaid.org/references/statements‐clarifications/). Among them, classification was determined in 78 samples: 12 samples were clade S; 16 samples were clade L; 2 samples were clade V; 38 samples were clade G; and 10 samples were clade GR. The detailed results of the 78 samples obtained by one‐step multiplex HRM method are shown in the Table S5 and the representative HRM curves are presented in Fig. 3 for five clades (S, L, V, G and GR clades).

Four samples could not be classified because some marker mutations failed to be identified using the one‐step multiplex HRM method. We speculated that the failure in detection resulted from low viral load in the samples, as their cycle threshold values were all > 35. Details of the four samples are listed in Table S6.

## Discussion

The rapid development of the COVID‐19 pandemic highlights shortcomings in the existing laboratory‐based testing strategy for SARS‐CoV‐2 diagnosis (Ganguli *et al*., 2020). Rapid, effective and reliable diagnostic methods are of paramount importance for combating the ongoing COVID‐19 pandemic since there is no antiviral drug to treat SARS‐CoV‐2 (Wang *et al*., 2020). Therefore, there is an immediate and urgent need for accurate, reliable and comprehensive diagnostic methods to detect SARS‐CoV‐2 infections (Feng *et al*., 2020; Sheridan, 2020; Wu *et al*., 2020; Yuan *et al*., 2020). Such detection assays are required not only in countries/regions that need to increase detection capacity as the pandemic grows but also in countries where surveillance efforts are needed to monitor the local/global distribution of different clades during the COVID‐19 pandemic. In view of this mandate, we have developed and evaluated a fast, easily extensible and comprehensive HRM‐based method for simultaneously detecting and typing SARS‐CoV‐2 directly from clinical samples. Our method integrates reverse transcription and multiplex PCR amplification followed by HRM analysis to facilitate the comprehensive diagnosis of SARS‐CoV‐2 in a one‐step, single‐tube reaction. This method is useful in rapid diagnosis of SARS‐CoV‐2 infection in COVID‐19 patients, asymptomatic carriers, suspected infections or close contact of confirmed cases. In addition, the method could also be used as a screening method to improve the surveillance of significant mutations, reducing the time to about 2 h.

During the global outbreak, several in‐house and commercial real‐time qRT‐PCR assays based on a variety of detection platforms have been developed, and some have received emergency approval from China’s National Medical Products Administration or Emergency Use Authorization by the U.S. Food and Drug Administration (Feng *et al*., 2020; Li and Ren, 2020; Tahamtan and Ardebili, 2020; Wu *et al*., 2020; Yuan *et al*., 2020; Yuan *et al*., 2020). These assays target different genes, including ORF1a, RNA‐dependent RNA polymerase, spike, E, and N, and have been proven highly specific for SARS‐CoV‐2 (Tang *et al*., 2020; Wu *et al*., 2020). However, some studies have reported that the detection rate of qRT‐PCR for COVID‐19 was as low as 30–60%, suggesting a high false negative rate (Afzal, 2020; Feng *et al*., 2020). To avoid false negative results, the one‐step multiplex HRM targets three genes (E, N and ORF1a) to increase the sensitivity and specificity and utilizes human RNase P for quality control of the RNA extraction. In real application scenarios, two structural genes (E and N) can be used to screen SARS‐CoV‐2 RNA in clinical specimens to reduce the false negative results caused by genetic mutations while the presence of SARS‐CoV‐2 will be further confirmed by the species‐specific target ORF1a. Using multiple targets, the one‐step multiplex HRM method has comparable analytical performance to real‐time qRT‐PCR. Furthermore, the HRM multiplex assay did not display decreased sensitivity compared with the singleplex assays (E‐HRM, N‐HRM and ORF1a‐HRM assays, data not shown). The probit analysis revealed that our method has sufficient sensitivity for detecting SARS‐CoV‐2 infection. With the LOD of 7.38–8.90 copies per reaction, our method is as sensitive as other real‐time qRT‐PCR‐based methods (Chan *et al*., 2020; Chu *et al*., 2020).

The one‐step multiplex HRM assay was expandable, rapid, economical and could provide classification information of SARS‐CoV‐2 prior to cumbersome sequencing, which is particularly suitable for resource limited situations. Currently, the comprehensive approach for genetic mutation monitoring is next‐generation sequencing (NGS); however, it is costly, time‐consuming and required expertise of bioinformatics (Ji *et al*., 2020). PCR coupled with Sanger sequencing has also been previously described as a method for detecting mutations (Xiu *et al*., 2020). However, due to the large number of mutation sites in SARS‐CoV‐2, analysing these sites by singleplex PCR requires very repetitive manual pipetting, therefore increase the risk of cross‐contamination. As far as we know, few methods were developed for analysing marker sites and typing SARS‐CoV −2. To overcome the limitations of NGS and Sanger sequencing, we developed the method based on HRM technology, which is multi‐target, cost‐effective and has less turnaround time. With these advantages, our method can accurately identify the phylogenetic clades of infected SARS‐CoV‐2 about 2 h, by simultaneously providing typing (S, L, V and G) and subtyping (GR, GV, GH and GRY) information. This strength can improve our understanding of the origin and spread of SARS‐CoV‐2, thus resulting in more timely management programmes aimed at controlling the further spread of COVID‐19.

Furthermore, our method is flexible, expandable and can be adapted as necessary. On the one hand, our assay can be separated into two panels for detecting the virus (assay 1) and screening mutations (assays 2–5) in SARS‐CoV‐2 to meet different diagnostic needs. On the other hand, as SARS‐CoV‐2 has evolved continuously with new mutations, novel important sites can be included to design a more comprehensive assay. Despite these advantages, one limitation of our study is that we only analysed a limited number of samples and sample types, which may be the reason why clades GH, GV and GRY were not found in our tested samples. Therefore, extended sample numbers and types, such as saliva and faeces, could potentially be included to further validate the clinical performance of the assay in the future. Due to the naturally expanding genetic diversity of SRAS‐CoV‐2, novel variants will be constantly emerging and spreading across countries, like the recently epidemic Delta variant (with the 478K, 681R mutation) in G clade, which has enhanced its ability of infection. Although our method is not as comprehensive as NGS, new assays can be easily added or replaced in a low‐cost manner when dealing with a novel epidemic variant. Therefore, due to the flexibility and scalability of our method, it proved to be a rapid and economical alternative to conventional sequencing‐based method in identifying mutation sites.

## Experimental procedures

### Complementary RNA (cRNA) standards

Concatenated fragments of the open reading frame 1a (ORF1a; nucleotides [Nt] 268 to 604), nucleocapsid (N; Nt 28927 to 29065)and envelope genes (E; Nt 26399 to 26457) of SARS‐CoV‐2 (GenBank accession no. NC_045512.2); a fragment of the human ribonuclease (RNase) P gene (Nt 1752 to 1948, GenBank accession no. NM_006413.5); and concatenated fragments containing each marker site and their respective flanking sequences were individually cloned into the pUC57 vector. The corresponding sequence inserted is displayed in the Table S1. The inserted fragment in each vector was amplified using primers containing the T7 promoter sequence (Table S2), and cRNA was generated by means of in vitro transcription. cRNA with fragments of the ORF1a, N and E genes of SARS‐CoV‐2 was defined as cRNA standard 1. cRNA with a fragment of the human RNase P gene was defined as cRNA standard 2. cRNA with the marker sites of 8782T, 28144C, 11083T, 26144T, 241T, 3037C, 23403A, 28882G, 25563T, 23063T, 23012T and 22227T was defined as cRNA standard 3, whereas cRNA with the marker sites of 8782C, 28144T, 11083G, 26144G, 241C, 3037T, 23403G, 28882A, 25563G, 23063A, 23012G and 22227C was defined as cRNA standard 4. RNA copy number was calculated from the concentrations of cRNA quantified using the Qubit^®^ RNA Assay Kit (Life Technologies, Carlsbad, CA, USA). The cRNA standards were diluted to concentrations of 0.5, 2.5, 5, 10, 25, 50, 500, 5000, 50 000 and 500 000 copies μl^−1^ in RNase‐free water to determine the limit of detection (LOD). Primer sets in each assay were used to simultaneously detect the corresponding target genes in LOD test.

### Specimens and extraction of viral RNA

Two hundred and ninety clinical samples were collected from patients with pneumonia or suspected SARS‐CoV‐2 infection, including 280 nasopharyngeal swabs and 10 sputum specimens. Viral RNA was extracted using the QIAamp Viral RNA Mini kit (Qiagen, Hilden, Germany) following the manufacturer’s instructions.

### Specificity testing panel

Nucleic acid from isolates and clinical samples that were positive for common pathogens in respiratory tract infections were used as a specificity panel to test for possible cross‐reactivity. The panel included 31 nasopharyngeal swabs positive for adenovirus, enterovirus, coronaviruses OC43, coronaviruses 229E, coronaviruses NL63, coronaviruses HKU1, MERS‐CoV, bocavirus, metapneumoviruses A and B, rhinovirus, influenza A H1N1, influenza A H3N2, influenza B, parainfluenza virus (PIV 1 to 4), respiratory syncytial viruses (RSV A and B), *Legionella pneumophila, Bordetella pertussis, Mycoplasma pneumoniae, Chlamydophila pneumoniae, Haemophilus influenzae, Staphylococcus aureus, Moraxella catarrhalis, Klebsiella pneumoniae, Pseudomonas aeruginosa, Acinetobacter baumannii, Escherichia coli* and two isolates of *Streptococcus pneumoniae* and *Neisseria meningitidis*.

### Primer design and optimization

Candidate primers for 16 target genes were designed using SnapGene software (GSL Biotech, Chicago, IL, USA), and their specificity was confirmed using the National Center for Biotechnology Information Primer‐BLAST tool (http://www.ncbi.nlm.nih.gov/tools/primer‐blast/). The theoretical melting temperature (*T*_m_) of the expected amplicons was calculated using OligoCalc (http://biotools.nubic.northwestern.edu/OligoCalc.html). G or GC tails were added to the 5’ end of some primers to increase the *T*_m_ of PCR products. Multiple pairs of primers for screening are shown in Table S4. The four cRNA standards were diluted to a concentration of 500 copies μl^−1^ and used to test different combinations of the candidate primers. Ideal combinations of primers should fulfil the requirement that the *T*_m_ ranges of different amplicons within one assay do not overlap. More precise *T*_m_ ranges were obtained from 11 repeat experiments. Ninety‐five percent confidence intervals (lower 95% confidence interval, upper 95% confidence interval) for all outcomes of each target were calculated and analysed as the results interpretation criteria of the one‐step multiplex HRM method. Moreover, the concentration of each primer was adjusted to make the height of the melting peak consistent.

### One‐step multiplex HRM

Figure 1 shows the whole experimental workflow and research design. Specifically, the one‐step multiplex HRM reaction included 10 μl 2× Reaction Mix (Invitrogen, Carlsbad, CA, USA), 1 μl 20× Evagreen (Biotium, Hayward, CA, USA), 1 μl SuperScript^®^ III RT/Platinum^®^ Taq Mix (Invitrogen), the corresponding volumes of primer pools for each assay (Table 1), 2 μl RNA template and nuclease‐free water for a total reaction volume of 20 μl. The one‐step multiplex HRM reaction conditions were as follows: reverse transcription PCR for 30 min at 55°C, followed by PCR activation for 2 min at 95°C, 30 cycles of amplification for 30 s at 94°C, 15 s at 53°C and 15 s signal collection at 68°C, and a final HRM step of 95°C for 15 s, 60°C for 1 min, 95°C for 15 s, continuous signal collection from 60 to 95°C at a rate of 0.025°C/s, and then cooling at 60°C. The one‐step multiplex HRM was performed using the Applied Biosystems^®^ QuantStudio™ 6 Flex Real‐Time PCR Instrument with fast 96‐well block and High Resolution Melt (Applied Biosystems Inc., Foster City, CA, USA). The melting curve and *T*_m_ values were analysed using High Resolution Melt module in QuantStudio™ Real‐Time PCR Software v1.2.

### qRT‐PCR and sequencing

Standard qRT‐PCR was used to detect the ORF1a and N genes of SARS‐CoV‐2 in clinical samples according to the manufacturer’s protocol (Anngeen Technologies, Beijing, China).

Based on the results of qRT‐PCR, cDNA of SARS‐CoV‐2 positive samples were synthesized using the SuperScript IV First‐Strand Synthesis System (Invitrogen). Five complete viral genomes were amplified from the above cDNA using the ARTIC amplicon sequencing protocol with the V3 primer sets (https://www.protocols.io/view/ncov‐2019‐sequencing‐protocol‐bbmuik6w) and then sequenced using a MinION sequencer (Oxford Nanopore Technologies, Oxford, UK). Data were analysed using the ARTIC bioinformatics protocol V1.1.0 (https://artic.network/ncov‐2019/ncov2019‐bioinformatics‐sop.html) and bases of the twelve marker sites were manually confirmed according to the BAM files of each sample. For the remaining SARS‐CoV‐2 positive samples, respective cDNA were used to amplify fragments containing the twelve marker sites using nested PCR with the FastStart High Fidelity PCR System (Roche, Basel, Switzerland), and the bases of the twelve marker sites were confirmed using Sanger sequencing. The primers used in nested PCR are listed in online Table S3.

### Statistical analysis

The LOD was calculated using probit analysis with SPSS Statistics software version 21.0 (SPSS Inc., Chicago, IL, USA). The *T*_m_ range (Results interpretation criteria) was performed by 95% confidence interval with GraphPad Prism 7.0 software (GraphPad Software, Inc., San Diego, CA).

## Conflict of interest

Junping Peng, Leshan Xiu and Liying Sun have a patent related to this article (202011572411.X). The authors have declared that no other competing financial interests exist.

## Ethical approval

All experiments were performed according to the ethical standards of the national research committee and approved by the Institutional Review Boards of the Institute of Pathogen Biology. All samples were obtained under approved ethical protocols and with informed consent from each patient.

## Author contributions

JP contributed to the development of the study design and the coordination of the execution of the study. LR and JP coordinated the study and reviewed drafts of the manuscript. LS drafted the study protocol, analysed the results and drafted the manuscript. LX and CZ conducted to the experiment and assisted in writing the manuscript. YX, YL and LZ helped to perform the experiments. All authors read and approved the final version of the paper.

## Supporting information


**Table S1**. The inserted sequence for synthetic RNA standard.
**Table S2**. The primer pairs containing T7 promoter for the *in vitro* transcription reaction.
**Table S3**. The primer pairs of nested PCR for conventional sequencing.
**Table S4**. The candidate primer sets of one step multiple HRM.
**Table S5**. Detailed results of 78 SARS‐CoV‐2 positive samples.
**Table S6**. Four SARS‐CoV‐2‐positive samples with partial marker sites identified.
