# Supplementary material for: Detection and classification of SARS‐CoV‐2 using high‐resolution melting analysis
Source: Microb Biotechnol. 2022 Mar 1;15(6):1883–94. doi: 10.1111/1751-7915.14027 (PMC9111094; doi:10.1111/1751-7915.14027)
Supplement: Supplementary file 1 — Table S1. The inserted sequence for synthetic RNA standard. Table S2. The primer pairs containing T7 promoter for the in vitro transcription reaction. Table S3. The primer pairs of nested PCR for conventional sequencing. Table S4. The candidate primer sets of one step multiple HRM. Table S5. Detailed results of 78 SARS‐CoV‐2 positive samples. Table S6. Four SARS‐CoV‐2‐positive samples with partial marker sites identified. [file MBT2-15-1883-s001.docx]

***Supplementary Material***

**Detection and classification of SARS-CoV-2 using high-resolution melting analysis**

Liying Sun ^1, 2, †^, Leshan Xiu ^1, 2, 3, †^, Chi Zhang ^1, 2, †^, Yan Xiao ^1, 2, 4^, Yamei Li ^1, 2^, Lulu Zhang ^1, 2^, Lili Ren ^1, 2, 4,^ *, Junping Peng ^1, 2,^ *

^1^ NHC Key Laboratory of Systems Biology of Pathogens, Institute of Pathogen Biology, Chinese Academy of Medical Sciences & Peking Union Medical College, Beijing, China

^2^ Key Laboratory of Respiratory Disease Pathogenomics, Chinese Academy of Medical Sciences and Peking Union Medical College, Beijing, China

^3^ School of Global Health, Chinese Center for Tropical Diseases Research, Shanghai Jiao Tong University School of Medicine

^4^ Christophe Merieux Laboratory, Institute of Pathogen Biology, Chinese Academy of Medical Sciences and Peking Union Medical College, Beijing, China.

^†^ These authors contributed equally in this study.

**Short running title:** Identification of SARS-CoV-2 by HRM

***Corresponding author**:

Junping Peng

Tel.: +86-10- 67878493

Fax: +86-10- 67878493

E-mail: pengjp@hotmail.com

Mailing address: No. 6 Rongjing Eastern Street, BDA, Beijing 100176, China.

Lili Ren

Tel.: +86-10-67855226

Fax: +86-10-67828516

E-mail: renliliipb@163.com.

Mailing address: No.9 Dong Dan San Tiao, Dongcheng District, Beijing 100730, P. R. China.

**Keywords**: COVID-19; SARS-CoV-2; clade classification; real-time PCR; high-resolution melting analysis

**Supplementary information**

**Supplemental Table S1.** The inserted sequence for synthetic RNA standard.

**Supplemental Table S2.** The primer pairs containing T7 promoter for the *in* *vitro* transcription reaction.

**Supplemental Table S3.** The primer pairs of nested PCR for conventional sequencing.

**Supplemental Table S4.** The candidate primer sets of one step multiple HRM.

**Supplemental Table S5.** Detailed results of 78 SARS-CoV-2 positive samples.

**Supplemental Table S6.** Four SARS-CoV-2-positive samples with partial marker sites identified.

Supplemental Table S1. The inserted sequence for synthetic RNA standard.

| cRNA standard | Target gene | Inserted sequence |
| --- | --- | --- |
| Standard 1 | ORF1a | AGGTTTCGTCCGGGTGTGACCGAAAGGTAAGATGGAGAGCCTTGTCCCTGGTTTCAACGAGAAAACACACGTCCAACTCAGTTTGCCTGTTTTACAGGTTCGCGACGTGCTCGTACGTGGCTTTGGAGACTCCGTGGAGGAGGTCTTATCAGAGGCACGTCAACATCTTAAAGATGGCACTTGTGGCTTAGTAGAAGTTGAAAAAGGCGTTTTGCCTCAACTTGAACAGCCCTATGTGTTCATCAAACGTTCGGATGCTCGAACTGCACCTCATGGTCATGTTATGGTTGAGCTGGTAGCAGAACTCGAAGGCATTCAGTACGGTCGTAGTGGTGAGACACTTGGTGTCCTTGTCCCTCATGTGGGCGAAATACCAGTGGCTTACCGCAAGGTTCTTCTTCGTAAGA |
|  | N | AACTTCTCCTGCTAGAATGGCTGGCAATGGCGGTGATGCTGCTCTTGCTTTGCTGCTGCTTGACAGATTGAACCAGCTTGAGAGCAAAATGTCTGGTAAAGGCCAACAACAACAAGGCCAAACTGTCACTAAGAAATCTGCTGCTGAGGCTTCTAAGAAGCCTCGGCAAAAACGTACTGCCACTAAAGCATACAATGTAACACAAGCTTTCGGCAGA |
|  | E | TGCAATATTGTTAACGTGAGTCTTGTAAAACCTTCTTTTTACGTTTACTCTCGTGTTAAAAATCTGAATTCTTCTAGAGTTCCTGATCTTCTGGTCTAAACGAACTAAATATTA |
| Standard 2 | RNase P | GTCTTTGAAATAGGATTCCTTACTTTTAGTTAGAAACCCCTAAAACGCTAATATTGATTGCAAAGCATCGGACTGAACCAACTTTGGAAATAATTTATTTTTATAATGGGATCATGTTAAGTAGAAGTAGCTTTTTATGCAAATACATGCATTTATGCAATATTAATGTAAGGGCTCTAAAACAATGGAGTAGAGCCAGAGGTATAACTGAATAAGAAATTTTTTTAAGCAAGAGAAAGACAACTGTTCTGCGGGT |
| Standard 3* | 28144 | AGAAAATCAGCACCTTTAATTGAATTGTGCGTGGATGAGGCTGGTTCTAAATCACCCATTCAGTACATCGATATCGGTAATTATACAGTTTCCTGTTTACCTTTTACAATTAATTGCCAGGAACCTAAATTGGGTAGTCTTGTAGTGCGTTGTTCGTTCTATG |
|  | 23403 | TGTTCTTTTGGTGGTGTCAGTGTTATAACACCAGGAACAAATACTTCTAACCAGGTTGCTGTTCTTTATCAGGATGTTAACTGCACAGAAGTCCCTGTTGCTATTCATGCAGATCAACTTACTCCTACTTGGCGTGTTTATTCTACAGGTTCTAATGTTTTTCAAACACGT |
|  | 26144 | AACATGTTACCTTCTTCATCTACAATAAAATTGTTGATGAGCCTGAAGAACATGTCCAAATTCACACAATCGACGTTTCATCCGGAGTTGTTAATCCAGTAATGGAACCAATTTATGATGAACCGACGACGACTACTAGCGTGCCTTTGTAAGCACAAGCTGATGAGTACGAACTTATGTACTCATTCGTTTCGGAAGAG |
|  | 3037 | GAGTGGAGTATGGCTACATACTACTTATTTGATGAGTCTGGTGAGTTTAAATTGGCTTCACATATGTATTGTTCTTTCTACCCTCCAGATGAGGATGAAGAAGAAGGTGATTGTGAAGAAGAAGAGTTTGAGCCATCAACTCAATATGAGTATGGTACTGAAGATGATTACCAA |
|  | 25563 | GATACCGATACAAGCCTCACTCCCTTTCGGATGGCTTATTGTTGGCGTTGCACTTCTTGCTGTTTTTCATAGCGCTTCCAAAATCATAACCCTCAAAAAGAGATGGCAACTAGCACTCTCCAAGGGTGTTCACTTTGTTTGCAACTTGCTGTTGTTGTT |
|  | 241 | CACGAGTAACTCGTCTATCTTCTGCAGGCTGCTTACGGTTTCGTCCGTGTTGCAGCCGATCATCAGCACATCTAGGTTTTGTCCGGGTGTGACCGAAAGGTAAGATGGAGAGCCTTGTCCCTGGTTTCAACGAGAAAACACACGTCCAACTCAGTTTGCCT |
|  | 11083 | ACACACCACTGGTTGTTACTCACAATTTTGACTTCACTTTTAGTTTTAGTCCAGAGTACTCAATGGTCTTTGTTCTTTTTTTTTTATGAAAATGCCTTTTTACCTTTTGCTATGGGTATTATTGCTATGTCTGCTTTTGCAATGATGTTTGTCAAACA |
|  | 8782 | AAGTGAAATCATAGGATACAAGGCTATTGATGGTGGTGTCACTCGTGACATAGCATCTACAGATACTTGTTTTGCTAACAAACATGCTGATTTTGACACATGGTTTAGCCAGCGTGGTGGTAGTTATACTAATGACAAAGCTTGCCCATTGATTGCTGCAGTCATAACAAGAGAAGTGGGTTTTGTCGTGC |
|  | 28882 | ATTGCCAAAAGGCTTCTACGCAGAAGGGAGCAGAGGCGGCAGTCAAGCCTCTTCTCGTTCCTCATCACGTAGTCGCAACAGTTCAAGAAATTCAACTCCAGGCAGCAGTAGGGGAACTTCTCCTGCTAGAATGGCTGGCAATGGCGGTGATGCTGCTCTTGCTTTGCTGCTGCTT |
|  | 23063 | AATGGTGTTGAAGGTTTTAATTGTTACTTTCCTTTACAATCATATGGTTTCCAACCCACTTATGGTGTTGGTTACCAACCATACAGAGTAGTAGTACTTTCTTTTGAACTTCTACATGCACCAGCA |
|  | 23012 | AACCTTTTGAGAGAGATATTTCAACTGAAATCTATCAGGCCGGTAGCACACCTTGTAATGGTGTTTAAGGTTTTAATTGTTACTTTCCTTTACAATCATATGGTTTCCAACCCACTAATGGTGTTGGTTACCAACCATACAGAG |
|  | 22227 | CTAAGCACACGCCTATTAATTTAGTGCGTGATCTCCCTCAGGGTTTTTCGGTTTTAGAACCATTGGTAGATTTGCCAATAGGTATTAACATCACTAGGTTTCAAACTTTACTTGC |
| Standard 4* | 28144 | AGAAAATCAGCACCTTTAATTGAATTGTGCGTGGATGAGGCTGGTTCTAAATCACCCATTCAGTACATCGATATCGGTAATTATACAGTTTCCTGTTCACCTTTTACAATTAATTGCCAGGAACCTAAATTGGGTAGTCTTGTAGTGCGTTGTTCGTTCTATG |
|  | 23403 | TGTTCTTTTGGTGGTGTCAGTGTTATAACACCAGGAACAAATACTTCTAACCAGGTTGCTGTTCTTTATCAGGGTGTTAACTGCACAGAAGTCCCTGTTGCTATTCATGCAGATCAACTTACTCCTACTTGGCGTGTTTATTCTACAGGTTCTAATGTTTTTCAAACACGT |
|  | 26144 | AACATGTTACCTTCTTCATCTACAATAAAATTGTTGATGAGCCTGAAGAACATGTCCAAATTCACACAATCGACGGTTCATCCGGAGTTGTTAATCCAGTAATGGAACCAATTTATGATGAACCGACGACGACTACTAGCGTGCCTTTGTAAGCACAAGCTGATGAGTACGAACTTATGTACTCATTCGTTTCGGAAGAG |
|  | 3037 | GAGTGGAGTATGGCTACATACTACTTATTTGATGAGTCTGGTGAGTTTAAATTGGCTTCACATATGTATTGTTCTTTTTACCCTCCAGATGAGGATGAAGAAGAAGGTGATTGTGAAGAAGAAGAGTTTGAGCCATCAACTCAATATGAGTATGGTACTGAAGATGATTACCAA |
|  | 25563 | GATACCGATACAAGCCTCACTCCCTTTCGGATGGCTTATTGTTGGCGTTGCACTTCTTGCTGTTTTTCAGAGCGCTTCCAAAATCATAACCCTCAAAAAGAGATGGCAACTAGCACTCTCCAAGGGTGTTCACTTTGTTTGCAACTTGCTGTTGTTGTT |
|  | 241 | CACGAGTAACTCGTCTATCTTCTGCAGGCTGCTTACGGTTTCGTCCGTGTTGCAGCCGATCATCAGCACATCTAGGTTTCGTCCGGGTGTGACCGAAAGGTAAGATGGAGAGCCTTGTCCCTGGTTTCAACGAGAAAACACACGTCCAACTCAGTTTGCCT |
|  | 11083 | ACACACCACTGGTTGTTACTCACAATTTTGACTTCACTTTTAGTTTTAGTCCAGAGTACTCAATGGTCTTTGTTCTTTTTTTTGTATGAAAATGCCTTTTTACCTTTTGCTATGGGTATTATTGCTATGTCTGCTTTTGCAATGATGTTTGTCAAACA |
|  | 8782 | AAGTGAAATCATAGGATACAAGGCTATTGATGGTGGTGTCACTCGTGACATAGCATCTACAGATACTTGTTTTGCTAACAAACATGCTGATTTTGACACATGGTTTAGTCAGCGTGGTGGTAGTTATACTAATGACAAAGCTTGCCCATTGATTGCTGCAGTCATAACAAGAGAAGTGGGTTTTGTCGTGC |
|  | 28882 | ATTGCCAAAAGGCTTCTACGCAGAAGGGAGCAGAGGCGGCAGTCAAGCCTCTTCTCGTTCCTCATCACGTAGTCGCAACAGTTCAAGAAATTCAACTCCAGGCAGCAGTAGAGGAACTTCTCCTGCTAGAATGGCTGGCAATGGCGGTGATGCTGCTCTTGCTTTGCTGCTGCTT |
|  | 23063 | AATGGTGTTGAAGGTTTTAATTGTTACTTTCCTTTACAATCATATGGTTTCCAACCCACTAATGGTGTTGGTTACCAACCATACAGAGTAGTAGTACTTTCTTTTGAACTTCTACATGCACCAGCA |
|  | 23012 | AACCTTTTGAGAGAGATATTTCAACTGAAATCTATCAGGCCGGTAGCACACCTTGTAATGGTGTTGAAGGTTTTAATTGTTACTTTCCTTTACAATCATATGGTTTCCAACCCACTAATGGTGTTGGTTACCAACCATACAGAG |
|  | 22227 | CTAAGCACACGCCTATTAATTTAGTGCGTGATCTCCCTCAGGGTTTTTCGGCTTTAGAACCATTGGTAGATTTGCCAATAGGTATTAACATCACTAGGTTTCAAACTTTACTTGC |

*The red bases denote marker sites.

Supplemental Table S2. The primer pairs containing T7 promoter for the *in* *vitro* transcription reaction.

| Primer | Sequence (5’ - 3’) * | Targets | Synthetic cRNA standard |
| --- | --- | --- | --- |
| ONE-F | TAATACGACTCACTATAGGGCGA AGGTTTCGTCCGGGTG | ORF 1a, N, E gene | cRNA standard 1 |
| ONE-R | TAATATTTAGTTCGTTTAGACCAGAAG |  |  |
| RNase P-F | TAATACGACTCACTATAGGGCGAGTCTTTGAAATAGGATTCCTTACTT | Human RNase P | cRNA standard 2 |
| RNase P-R | ACCCGCAGAACAGTTGT |  |  |
| Mutation sits-F | TAATACGACTCACTATAGGGCGA AGAAAATCAGCACCTTTAATTGAATT | 28144 ,23403, 26144, 3037, 25563, 241, 11082, 8782, 28882, 23063, 23012, and 22227 | cRNA standard 3 and 4 |
| Mutation sites-R | GCAAGTAAAGTTTGAAACCTAGTG |  |  |

* The underlined bases denote T7 promoter sequence (TAATACGACTCACTATAGGGCGA).

Supplemental Table S3. The primer pairs of nested PCR for conventional sequencing.

| Targets | Primer | Sequence (5’-3’) |
| --- | --- | --- |
| ORF8 | 28144-Nested PCR-F1 | AGTCATGTACTCAACATCAACCATATG |
|  | 28144-Nested PCR-R1 | CACTGCGTTCTCCATTCTGG |
|  | 28144-Nested PCR-F2 | GGTATATTAGAGTAGGAGCTAGAAAATCAGCACC |
|  | 28144-Nested PCR-R2 | GGGGTCCATTATCAGACATTTTAGTTTGTTC |
| S | 23403-Nested PCR-F1 | TGAGTCTAACAAAAAGTTTCTGCCT |
|  | 23403-Nested PCR-R1 | ATGATGGATTGACTAGCTACACTACG |
|  | 23403-Nested PCR-F2 | CAGACACTTGAGATTCTTGACATTACACCAT |
|  | 23403-Nested PCR-R2 | ATGAGTTGTTGACATGTTCAGCCC |
| ORF3a | 26144-Nested PCR-F1 | AGTCCTATTTCTGAACATGACTACCA |
|  | 26144-Nested PCR-R1 | GGCTAGTGTAACTAGCAAGAATACC |
|  | 26144-Nested PCR-F2 | GTGTTGTATTACACAGTTACTTCACTTCAGACT |
|  | 26144-Nested PCR-R2 | TCGTACTCATCAGCTTGTGCTTACAAA |
| ORF1ab | 3037-Nested PCR-F1 | ACTTAATGAGAAGTGCTCTGCC |
|  | 3037-Nested PCR-R1 | TCTTGTTGACCAACAGTTTGTTGA |
|  | 3037-Nested PCR-F2 | TGGAGTATGGCTACATACTACTTATTTGATGAGT |
|  | 3037-Nested PCR-R2 | CATCTAACCAATCTTCTTCTTGCTCTTCTTCA |
| ORF3a | 25563-Nested PCR-F1 | ATAAACGAACTTATGGATTTGTTTATGAGAATC |
|  | 25563-Nested PCR-R1 | TTTGGAACGGCATTTCCAGC |
|  | 25563-Nested PCR-F2 | TCAAGGATGCTACTCCTTCAGATTTTGTTC |
|  | 25563-Nested PCR-R2 | GCTTCAAGGCCAGCAGCAAC |
| 5’ UTR | 241-Nested PCR-F1 | GTTCTCTAAACGAACTTTAAAATCTGTGTG |
|  | 241-Nested PCR-R1 | TTGATGAACACATAGGGCTGTTC |
|  | 241-Nested PCR-F2 | CTAATTACTGTCGTTGACAGGACACGAG |
|  | 241-Nested PCR-R2 | AGACCTCCTCCACGGAGTCT |
| ORF1ab | 11083-Nested PCR-F1 | AGAATTACTGCAAAATGGTATGAATGGA |
|  | 11083-Nested PCR-R1 | GTATCAACCATATCCAACCATGTCA |
|  | 11083-Nested PCR-F2 | TTAGTCCAGAGTACTCAATGGTCTTTGT |
|  | 11083-Nested PCR-R2 | ACAAACAGAGAAATGCATGCTTATGTTTG |
| ORF1ab | 8782-Nested PCR-F1 | GTTAATTAAAGTTACACTTGTGTTCCTTTTTG |
|  | 8782-Nested PCR-R1 | GCTGATGTTGCAAAGTCAGTG |
|  | 8782-Nested PCR-F2 | AATAACACCTGTTCATGTCATGTCTAAACAT |
|  | 8782-Nested PCR-R2 | CATTAGTTGTGCGTAATATCGTGCCAG |
| N | 28882-Nested PCR-F1 | AGCCTTGAATACACCAAAAGATCA |
|  | 28882-Nested PCR-R1 | CTGCCGAAAGCTTGTGTTACA |
|  | 28882-Nested PCR-F2 | TGCTAACAATGCTGCAATCGTGC |
|  | 28882-Nested PCR-R2 | CTTGTTGTTGTTGGCCTTTACCAGAC |
| S | 23063-Nested-F1 | ACCTTTTGAGAGAGATATTTCAAC |
|  | 23063-Nested-R1 | CCAGCAACTGTTTGTGGAC |
|  | 23063-Nested-F2 | CAGGCCGGTAGCACAC |
|  | 23063-Nested-R2 | GTTACCAACCATACAGAGTAGT |
| S | 22227-Nested-F1 | CTTAGGGAATTTGTGTTTAAGAATATT |
|  | 22227-Nested-R1 | GCAAGTAAAGTTTGAAACCTAGTG |
|  | 22227-Nested-F2 | GCACTAAATTAATAGGCGTGTG |
|  | 22227-Nested-R2 | GGCAAATCTACCAATGGTTCT |
| S | 23012-Nested-F1 | AATCTTGATTCTAAGGTTGGTGG |
|  | 23012-Nested-R1 | ACCATACAGAGTAGTAGTACTTT |
|  | 23012-Nested-F2 | CTATCAGGCCGGTAGCA |
|  | 23012-Nested-R2 | GGGTTGGAAACCATATGATTGT |

Supplemental Table S4. The candidate primer sets of one-step multiplex HRM.

| Panel | Targets | Primer | Sequence (5’ - 3’) * |
| --- | --- | --- | --- |
| Assay 1 | ORF 1a | ORF1ab-F | GGAGAGCCTTGTCCCTGGTTTCAACGAG |
|  |  | ORF1ab-R | TTCGCCCACATGAGGGACAAGGACA |
|  |  | ORF1ab-F1 | TTTAGCCAGCGTGGTGGTAGTTAT |
|  |  | ORF1ab-R1 | GGCACGACAAAACCCACTTCTC |
|  |  | ORF1ab-F2 | TGTTCCTCGGAACTTGTCGG |
|  |  | ORF1ab-R2 | CTCCAAGCAGGGTTACGTGT |
|  | N | N -F | TCTTGCTTTGCTGCTGCTTGACAGA |
|  |  | N-R | GCAGTACGTTTTTGCCGAGGCTT |
|  |  | N -F1 | ACCCGCAATCCTGCTAACAATG |
|  |  | N-R1 | CAAGCAGCAGCAAAGCAAGAGC |
|  |  | N -F2 | GCAGTCAAGCCTCTTCTCGTTCCT |
|  |  | N-R2 | TCAAGCAGCAGCAAAGCAAGAGCA |
|  | E | E-F | TAAAACCTTCTTTTTACGTTTACTCTCG |
|  |  | E-R | GGAACTCTAGAAGAATTCAGATTTTTAAC |
|  |  | E-F1 | TTTAGCCAGCGTGGTGGTAGTTAT |
|  |  | E-R1 | GGCACGACAAAACCCACTTCTC |
|  |  | E-F2 | TTCGTTTCGGAAGAGACAGGTACGT |
|  |  | E-R2 | GCGCAGTAAGGATGGCTAGTGTAAC |
|  | RNase P | Rnase P-F | GCAAAGCATCGGACTGAACC |
|  |  | Rnase P-R | ACCCGCAGAACAGTTGTCTT |
|  |  | Rnase P-F1 | CAGTGAAGAAACCTCGGCCATCAGAA |
|  |  | Rnase P-R1 | AAGGGAAGAAGGGAGTGCTGACAGA |
|  |  | Rnase P-F2 | CATGGCGGTGTTTGCAGATTTGG |
|  |  | Rnase P-R2 | TGAATAGCCAAGGTGAGCGGC |
| Assay 2 | ORF8 | 28144-F | GTTCTAAATCACCCATTCAGTACAT |
|  |  | 28144-R | CCAATTTAGGTTCCTGGCAATTA |
|  |  | 28144-F1 | TGAGGCTGGTTCTAAATCACC |
|  |  | 28144-R1 | ACCCAATTTAGGTTCCTGGCAA |
|  |  | 28144-F2 | TGTGCGTGGATGAGGC |
|  |  | 28144-R2 | CAATTTAGGTTCCTGGCAATTAATTGT |
|  | S | 23403-F | CACCAGGAACAAATACTTCTAACCAGG |
|  |  | 23403-R | GTAGAATAAACACGCCAAGTAGGAGTAAG |
|  |  | 23403-F | CTCTTCTTCTTCACAATCACCTTCTTCTT |
|  |  | 23403-R | CTCTTCTTCTTCACAATCACCTTCTTCTT |
|  |  | 23403-F | GGAACAAATACTTCTAACCAGGTTG |
|  |  | 23403-R | GCCAAGTAGGAGTAAGTTGATCTG |
|  | ORF3a | 26144-F | GCGCGCGTTGATGAGCCTGAAGAACATGTC |
|  |  | 26144-R | GCGCGCGCTTGTGCTTACAAAGGCACG |
|  |  | 26144-F | GCGCGCGCCTGAAGAACATGTCCAAATTCAC |
|  |  | 26144-R | GCGCGCGGCACGCTAGTAGTCGTCG |
|  |  | 26144-F | ATGTCCAAATTCACACAATCGAC |
|  |  | 26144-R | AGTAGTCGTCGTCGGTTC |
| Assay 3 | ORF1ab | 3037-F | CTGGTGAGTTTAAATTGGCTTCACATATG |
|  |  | 3037-R | TCAAACTCTTCTTCTTCACAATCACCTTC |
|  |  | 3037-F1 | GAGTTTAAATTGGCTTCACATATGTATTGTTCTT |
|  |  | 3037-R1 | CTCTTCTTCTTCACAATCACCTTCTTCTT |
|  |  | 3037-F2 | GGTGAGTTTAAATTGGCTTCACA |
|  |  | 3037-R2 | TTCTTCACAATCACCTTCTTCTTCATC |
|  | ORF3a | 25563-F | GCTTATTGTTGGCGTTGCACTTCT |
|  |  | 25563-R | CTTGGAGAGTGCTAGTTGCCATCTC |
|  |  | 25563-F1 | GCGCGCGTTCGCGCTACTGCAACG |
|  |  | 25563-R1 | GCGCGCGCAAGTTGCAAACAAAGTGAACAC |
|  |  | 25563-F2 | GCGCCGGCGCTACTGCAACGATACCG |
|  |  | 25563-R2 | GCGCCGCCTTGGAGAGTGCTAGTTGCC |
|  | 5’ UTR | 241-F | TCGTCCGTGTTGCAGCC |
|  |  | 241-R | CCAGGGACAAGGCTCTCCA |
|  |  | 241-F1 | CCGTGTTGCAGCCGATCA |
|  |  | 241-R1 | AAGGCTCTCCATCTTACCTTTCGG |
|  |  | 241-F2 | GTTGCAGCCGATCATCAG |
|  |  | 241-R2 | CCAGGGACAAGGCTCTCC |
| Assay 4 | ORF1ab | 11083-F | GCGCAGAGTACTCAATGGTCTTTGTTCTTTTTTT |
|  |  | 11083-R | GCGCATAGCAAAAGGTAAAAAGGCATTTTCAT |
|  |  | 11083-F1 | TTAGTCCAGAGTACTCAATGGTCTTTGT |
|  |  | 11083-R1 | CATAGCAAAAGGTAAAAAGGCATTTTCAT |
|  |  | 11083-F2 | TAGTTTTAGTCCAGAGTACTCAATGG |
|  |  | 11083-R2 | CCCATAGCAAAAGGTAAAAAGGCAT |
|  | ORF1ab | 8782-F | ACTCGTGACATAGCATCTACAG |
|  |  | 8782-R | TGCAGCAATCAATGGGCAA |
|  |  | 8782-F1 | TGATGGTGGTGTCACTCG |
|  |  | 8782-R1 | TCTCTTGTTATGACTGCAGCA |
|  |  | 8782-F2 | TACTTGTTTTGCTAACAAACATGCTG |
|  |  | 8782-R2 | ACAAAACCCACTTCTCTTGTTATGA |
|  | N | 28882-F | GGCGGCAGTCAAGCC |
|  |  | 28882-R | CCGCCATTGCCAGCC |
|  |  | 28882-F1 | CATAGCAAAAGGTAAAAAGGCATTTTCAT |
|  |  | 28882-R1 | CCGCCATTGCCAGCC |
|  |  | 28882-F2 | CCGCCATTGCCAGCC |
|  | S | 23063-F1 | CTTTCCTTTACAATCATATGGTTTCCAACCC |
|  |  | 23063-R1 | ACTACTACTCTGTATGGTTGGTAACCAACA |
|  | S | 22227-F1 | GCACACGCCTATTAATTTAGTGCGT |
|  |  | 22227-R1 | CCTATTGGCAAATCTACCAATGGTTCT |
| Assay 5 | S | 23063-F1 | CCTTTACAATCATATGGTTTCCAA |
|  |  | 23063-R1 | GTATGGTTGGTAACCAACACCA |
|  |  | 23063-F2 | CCTTTACAATCATATGGTTT |
|  |  | 23063-R2 | CTCTGTATGGTTGGTAACCAA |
|  | S | 23012-F1 | CGCGGTAGCACACCTTGTAATGGTG |
|  |  | 23012-R1 | GGGTTGGAAACCATATGATT |
|  |  | 23012-F2 | CCGCGGTAGCACACCTTGT |
|  |  | 23012-R2 | AAACCATATGATTGTAAAGGAAAG |
|  | S | 22227-F1 | GCGCGCAGTGCGTGATCTCCCTCAG |
|  |  | 22227-R1 | CTATTGGCAAATCTACCAATGGTTCT |
|  |  | 22227-F2 | GCGCGCGCGCAGTGCGTGATCTCCCTCAG |
|  |  | 22227-R2 | CCTATTGGCAAATCTACCAA |

* G or GC tails added to the 5’ end of primers were underlined.

Supplemental Table S5. Detailed results of 78 SARS-CoV-2 positive samples.

| Sample number | 8782 | 28144 | 241 | 3037 | 23403 | 11083 | 26144 | 25563 | 28882 | 22227 | 23063 | 23012 | Clades |
| --- | --- | --- | --- | --- | --- | --- | --- | --- | --- | --- | --- | --- | --- |
|  | Base (Tm value) | | | | | | | | | | | |  |
| 1 | T(78.57) | C(75.64) | C(83.75) | C(75.68) | A(79.12) | G(75.25) | G(83.92) | G(79.95) | G(83.92) | C(83.71) | A(74.92) | G(79.99) | S |
| 2 | T(78.55) | C(75.64) | C(83.80) | C(75.61) | A(79.12) | G(75.25) | G(83.95) | G(79.96) | G(83.93) | C(83.73) | A(74.98) | G(79.98) | S |
| 3 | T(78.56) | C(75.64) | C(83.76) | C(75.61) | A(79.16) | G(75.24) | G(83.96) | G(79.96) | G(83.96) | C(83.76) | A(74.95) | G(80.02) | S |
| 4 | T(78.51) | C(75.64) | C(83.74) | C(75.63) | A(79.15) | G(75.24) | G(83.97) | G(79.94) | G(83.97) | C(83.75) | A(74.96) | G(80.05) | S |
| 5 | T(78.57) | C(75.64) | C(83.75) | C(75.63) | A(79.16) | G(75.24) | G(83.96) | G(79.98) | G(83.95) | C(83.77) | A(74.92) | G(79.99) | S |
| 6 | T(78.55) | C(75.62) | C(83.75) | C(75.63) | A(79.15) | G(75.24) | G(83.97) | G(79.95) | G(83.96) | C(83.76) | A(74.97) | G(80.06) | S |
| 7 | T(78.56) | C(75.62) | C(83.77) | C(75.67) | A(79.15) | G(75.26) | G(83.96) | G(79.94) | G(83.93) | C(83.77) | A(74.91) | G(80.08) | S |
| 8 | T(78.57) | C(75.68) | C(83.80) | C(75.66) | A(79.15) | G(75.26) | G(83.94) | G(79.96) | G(83.93) | C(83.76) | A(74.98) | G(80.09) | S |
| 9 | T(78.54) | C(75.64) | C(83.77) | C(75.62) | A(79.16) | G(75.29) | G(83.97) | G(79.96) | G(83.97) | C(83.73) | A(74.91) | G(80.05) | S |
| 10 | T(78.52) | C(75.64) | C(83.77) | C(75.64) | A(79.16) | G(75.29) | G(83.92) | G(79.97) | G(83.95) | C(83.73) | A(74.96) | G(80.08) | S |
| 11 | T(78.51) | C(75.69) | C(83.76) | C(75.61) | A(79.16) | G(75.26) | G(83.92) | G(79.94) | G(83.96) | C(83.77) | A(74.97) | G(80.06) | S |
| 12 | T(78.53) | C(75.64) | C(83.77) | C(75.61) | A(79.12) | G(75.29) | G(83.92) | G(79.96) | G(83.96) | C(83.76) | A(74.98) | G(80.02) | S |
| 13 | C(78.92) | T(74.83) | C(83.74) | C(75.62) | A(79.12) | G(75.23) | G(83.95) | G(79.95) | G(83.97) | C(83.76) | A(74.96) | G(80.02) | L |
| 14 | C(78.96) | T(74.85) | C(83.74) | C(75.62) | A(79.12) | G(75.23) | G(83.94) | G(79.99) | G(83.95) | C(83.76) | A(74.92) | G(80.03) | L |
| 15 | C(78.94) | T(74.84) | C(83.74) | C(75.64) | A(79.15) | G(75.23) | G(83.92) | G(79.99) | G(83.96) | C(83.76) | A(74.92) | G(80.05) | L |
| 16 | C(78.94) | T(74.89) | C(83.75) | C(75.62) | A(79.15) | G(75.23) | G(83.95) | G(79.95) | G(83.97) | C(83.76) | A(74.91) | G(80.05) | L |
| 17 | C(78.92) | T(74.84) | C(83.74) | C(75.64) | A(79.15) | G(75.24) | G(83.94) | G(79.94) | G(83.97) | C(83.76) | A(74.92) | G(80.02) | L |
| 18 | C(78.92) | T(74.84) | C(83.79) | C(75.61) | A(79.14) | G(75.24) | G(83.96) | G(79.96) | G(83.92) | C(83.73) | A(74.93) | G(80.02) | L |
| 19 | C(78.94) | T(74.83) | C(83.79) | C(75.63) | A(79.16) | G(75.26) | G(83.95) | G(79.98) | G(83.96) | C(83.75) | A(74.93) | G(80.06) | L |
| 20 | C(78.95) | T(74.84) | C(83.77) | C(75.68) | A(79.15) | G(75.29) | G(83.92) | G(79.94) | G(83.96) | C(83.77) | A(74.92) | G(80.02) | L |
| 21 | C(78.95) | T(74.84) | C(83.77) | C(75.68) | A(79.15) | G(75.24) | G(83.96) | G(79.94) | G(83.96) | C(83.77) | A(74.91) | G(80.02) | L |
| 22 | C(78.95) | T(74.84) | C(83.76) | C(75.66) | A(79.13) | G(75.24) | G(83.95) | G(79.95) | G(83.96) | C(83.76) | A(74.93) | G(80.05) | L |
| 23 | C(78.92) | T(74.86) | C(83.75) | C(75.64) | A(79.16) | G(75.24) | G(83.94) | G(79.96) | G(83.95) | C(83.75) | A(74.97) | G(80.04) | L |
| 24 | C(78.92) | T(74.84) | C(83.75) | C(75.62) | A(79.14) | G(75.26) | G(83.94) | G(79.94) | G(83.96) | C(83.77) | A(74.92) | G(80.06) | L |
| 25 | C(78.95) | T(74.83) | C(83.76) | C(75.63) | A(79.15) | G(75.26) | G(83.94) | G(79.94) | G(83.97) | C(83.77) | A(74.92) | G(80.02) | L |
| 26 | C(78.94) | T(74.83) | C(83.77) | C(75.63) | A(79.16) | G(75.28) | G(83.92) | G(79.94) | G(83.95) | C(83.77) | A(74.96) | G(80.02) | L |
| 27 | C(78.93) | T(74.84) | C(83.74) | C(75.66) | A(79.16) | G(75.29) | G(83.92) | G(79.96) | G(83.93) | C(83.77) | A(74.96) | G(80.02) | L |
| 28 | C(78.94) | T(74.84) | C(83.77) | C(75.61) | A(79.15) | G(75.29) | G(83.92) | G(79.98) | G(83.96) | C(83.73) | A(74.91) | G(80.03) | L |
| 29 | C(78.93) | T(74.86) | C(83.74) | C(75.62) | A(79.15) | T(74.66) | T(83.26) | G(79.95) | G(83.92) | C(83.73) | A(74.95) | G(80.08) | V |
| 30 | C(78.92) | T(74.84) | C(83.75) | C(75.62) | A(79.13) | T(74.66) | T(83.27) | G(79.99) | G(83.94) | C(83.76) | A(74.98) | G(80.05) | V |
| 31 | C(78.93) | T(74.83) | T(83.15) | T(75.23) | G(79.58) | G(75.23) | G(83.95) | G(79.95) | G(83.92) | C(83.72) | A(74.95) | G(80.05) | G |
| 32 | C(78.93) | T(74.84) | T(83.16) | T(75.26) | G(79.56) | G(75.26) | G(83.96) | G(79.96) | G(83.93) | C(83.73) | A(74.95) | G(80.05) | G |
| 33 | C(78.93) | T(74.83) | T(83.16) | T(75.28) | G(79.57) | G(75.26) | G(83.96) | G(79.94) | G(83.96) | C(83.72) | A(74.91) | G(80.04) | G |
| 34 | C(78.93) | T(74.85) | T(83.15) | T(75.29) | G(79.56) | G(75.28) | G(83.95) | G(79.98) | G(83.96) | C(83.72) | A(74.92) | G(80.04) | G |
| 35 | C(78.96) | T(74.83) | T(83.15) | T(75.26) | G(79.57) | G(75.23) | G(83.92) | G(79.96) | G(83.97) | C(83.75) | A(74.92) | G(80.02) | G |
| 36 | C(78.92) | T(74.85) | T(83.16) | T(75.29) | G(79.56) | G(75.28) | G(83.92) | G(79.94) | G(83.95) | C(83.74) | A(74.92) | G(80.02) | G |
| 37 | C(78.93) | T(74.83) | T(83.16) | T(75.28) | G(79.57) | G(75.24) | G(83.94) | G(79.94) | G(83.96) | C(83.74) | A(74.96) | G(80.02) | G |
| 38 | C(78.93) | T(74.88) | T(83.15) | T(75.23) | G(79.56) | G(75.24) | G(83.95) | G(79.94) | G(83.96) | C(83.74) | A(74.92) | G(80.03) | G |
| 39 | C(78.96) | T(74.88) | T(83.16) | T(75.26) | G(79.56) | G(75.28) | G(83.95) | G(79.99) | G(83.97) | C(83.71) | A(74.92) | G(80.02) | G |
| 40 | C(78.96) | T(74.85) | T(83.19) | T(75.24) | G(79.59) | G(75.29) | G(83.95) | G(79.94) | G(83.97) | C(83.77) | A(74.98) | G(80.02) | G |
| 41 | C(78.98) | T(74.85) | T(83.15) | T(75.28) | G(79.52) | G(75.29) | G(83.96) | G(79.94) | G(83.97) | C(83.73) | A(74.92) | G(80.05) | G |
| 42 | C(78.96) | T(74.86) | T(83.15) | T(75.29) | G(79.53) | G(75.30) | G(83.92) | G(79.96) | G(83.95) | C(83.76) | A(74.98) | G(80.02) | G |
| 43 | C(78.96) | T(74.84) | T(83.16) | T(75.29) | G(79.56) | G(75.29) | G(83.92) | G(79.96) | G(83.96) | C(83.77) | A(74.95) | G(80.02) | G |
| 44 | C(78.95) | T(74.83) | T(83.17) | T(75.23) | G(79.52) | G(75.29) | G(83.97) | G(79.98) | G(83.97) | C(83.77) | A(74.98) | G(80.03) | G |
| 45 | C(78.94) | T(74.85) | T(83.16) | T(75.26) | G(79.54) | G(75.26) | G(83.94) | G(79.95) | G(83.97) | C(83.77) | A(74.97) | G(80.05) | G |
| 46 | C(78.95) | T(74.85) | T(83.19) | T(75.26) | G(79.51) | G(75.24) | G(83.96) | G(79.95) | G(83.97) | C(83.75) | A(74.92) | G(80.08) | G |
| 47 | C(78.97) | T(74.86) | T(83.17) | T(75.28) | G(79.52) | G(75.26) | G(83.92) | G(79.94) | G(83.96) | C(83.76) | A(74.91) | G(80.07) | G |
| 48 | C(78.96) | T(74.88) | T(83.16) | T(75.28) | G(79.52) | G(75.24) | G(83.92) | G(79.95) | G(83.96) | C(83.74) | A(74.93) | G(80.08) | G |
| 49 | C(78.96) | T(74.89) | T(83.15) | T(75.29) | G(79.53) | G(75.25) | G(83.93) | G(79.94) | G(83.97) | C(83.77) | A(74.92) | G(80.05) | G |
| 50 | C(78.99) | T(74.88) | T(83.15) | T(75.29) | G(79.58) | G(75.26) | G(83.92) | G(79.96) | G(83.96) | C(83.71) | A(74.98) | G(80.06) | G |
| 51 | C(78.97) | T(74.88) | T(83.16) | T(75.26) | G(79.52) | G(75.29) | G(83.96) | G(79.96) | G(83.92) | C(83.76) | A(74.95) | G(80.06) | G |
| 52 | C(78.97) | T(74.84) | T(83.19) | T(75.23) | G(79.52) | G(75.29) | G(83.92) | G(79.96) | G(83.93) | C(83.74) | A(74.97) | G(80.04) | G |
| 53 | C(78.96) | T(74.89) | T(83.15) | T(75.23) | G(79.56) | G(75.30) | G(83.92) | G(79.96) | G(83.96) | C(83.71) | A(74.90) | G(80.04) | G |
| 54 | C(78.95) | T(74.85) | T(83.18) | T(75.23) | G(79.56) | G(75.24) | G(83.94) | G(79.98) | G(83.96) | C(83.71) | A(74.95) | G(80.01) | G |
| 55 | C(78.96) | T(74.86) | T(83.15) | T(75.24) | G(79.53) | G(75.24) | G(83.95) | G(79.96) | G(83.97) | C(83.71) | A(74.96) | G(80.02) | G |
| 56 | C(78.97) | T(74.86) | T(83.16) | T(75.28) | G(79.53) | G(75.24) | G(83.96) | G(79.96) | G(83.97) | C(83.76) | A(74.96) | G(80.01) | G |
| 57 | C(78.97) | T(74.84) | T(83.19) | T(75.26) | G(79.56) | G(75.24) | G(83.92) | G(79.94) | G(83.97) | C(83.77) | A(74.90) | G(80.03) | G |
| 58 | C(78.96) | T(74.85) | T(83.17) | T(75.23) | G(79.52) | G(75.29) | G(83.94) | G(79.96) | G(83.97) | C(83.77) | A(74.92) | G(80.04) | G |
| 59 | C(78.97) | T(74.84) | T(83.17) | T(75.23) | G(79.52) | G(75.24) | G(83.94) | G(79.96) | G(83.96) | C(83.75) | A(74.91) | G(80.04) | G |
| 60 | C(78.96) | T(74.88) | T(83.17) | T(75.26) | G(79.56) | G(75.23) | G(83.97) | G(79.94) | G(83.97) | C(83.71) | A(74.96) | G(80.08) | G |
| 61 | C(78.96) | T(74.88) | T(83.19) | T(75.28) | G(79.53) | G(75.23) | G(83.97) | G(79.94) | G(83.95) | C(83.71) | A(74.92) | G(80.05) | G |
| 62 | C(78.96) | T(74.85) | T(83.17) | T(75.26) | G(79.56) | G(75.26) | G(83.96) | G(79.94) | G(83.96) | C(83.76) | A(74.97) | G(80.08) | G |
| 63 | C(78.96) | T(74.89) | T(83.17) | T(75.28) | G(79.54) | G(75.24) | G(83.94) | G(79.94) | G(83.96) | C(83.77) | A(74.95) | G(80.09) | G |
| 64 | C(78.93) | T(74.86) | T(83.17) | T(75.23) | G(79.58) | G(75.24) | G(83.92) | G(79.97) | G(83.97) | C(83.75) | A(74.93) | G(80.08) | G |
| 65 | C(78.96) | T(74.85) | T(83.16) | T(75.28) | G(79.58) | G(75.24) | G(83.97) | G(79.97) | G(83.97) | C(83.75) | A(74.96) | G(80.08) | G |
| 66 | C(78.97) | T(74.89) | T(83.16) | T(75.29) | G(79.52) | G(75.28) | G(83.92) | G(79.97) | G(83.92) | C(83.76) | A(74.91) | G(80.01) | G |
| 67 | C(78.96) | T(74.88) | T(83.16) | T(75.23) | G(79.52) | G(75.24) | G(83.95) | G(79.97) | G(83.96) | C(83.75) | A(74.98) | G(80.06) | G |
| 68 | C(78.99) | T(74.88) | T(83.19) | T(75.23) | G(79.52) | G(75.24) | G(83.95) | G(79.98) | G(83.92) | C(83.77) | A(74.97) | G(80.02) | G |
| 69 | C(78.92) | T(74.84) | T(83.16) | T(75.23) | G(79.59) | G(75.24) | G(83.96) | G(79.96) | A(83.22) | C(83.74) | A(74.95) | G(80.04) | GR |
| 70 | C(78.93) | T(74.84) | T(83.16) | T(75.26) | G(79.56) | G(75.23) | G(83.95) | G(79.98) | A(83.22) | C(83.72) | A(74.92) | G(80.05) | GR |
| 71 | C(78.93) | T(74.86) | T(83.18) | T(75.28) | G(79.56) | G(75.25) | G(83.95) | G(80.04) | A(83.26) | C(83.74) | A(74.91) | G(80.04) | GR |
| 72 | C(78.93) | T(74.89) | T(83.16) | T(75.24) | G(79.54) | G(75.25) | G(83.96) | G(79.96) | A(83.23) | C(83.74) | A(74.96) | G(80.03) | GR |
| 73 | C(78.96) | T(74.85) | T(83.17) | T(75.28) | G(79.52) | G(75.23) | G(83.92) | G(79.98) | A(83.23) | C(83.74) | A(74.96) | G(80.06) | GR |
| 74 | C(78.96) | T(74.85) | T(83.16) | T(75.29) | G(79.52) | G(75.26) | G(83.92) | G(79.96) | A(83.22) | C(83.75) | A(74.97) | G(80.04) | GR |
| 75 | C(78.96) | T(74.86) | T(83.16) | T(75.29) | G(79.51) | G(75.28) | G(83.95) | G(79.97) | A(83.26) | C(83.72) | A(74.95) | G(80.02) | GR |
| 76 | C(78.99) | T(74.88) | T(83.16) | T(75.29) | G(79.53) | G(75.26) | G(83.92) | G(79.97) | A(83.26) | C(83.74) | A(74.91) | G(80.04) | GR |
| 77 | C(78.99) | T(74.88) | T(83.15) | T(75.23) | G(79.52) | G(75.29) | G(83.92) | G(79.96) | A(83.27) | C(83.71) | A(74.95) | G(80.01) | GR |
| 78 | C(78.95) | T(74.88) | T(83.18) | T(75.29) | G(79.58) | G(75.28) | G(83.94) | G(79.95) | A(83.29) | C(83.74) | A(74.96) | G(80.05) | GR |

Supplemental Table S6. Four SARS-CoV-2-positive samples with partial marker sites identified

| Sample number | Twelve marker sites | | | | | | | | | | | |
| --- | --- | --- | --- | --- | --- | --- | --- | --- | --- | --- | --- | --- |
|  | 28144 | 8782 | 26144 | 241 | 23403 | 3037 | 28882 | 11083 | 25563 | 22227 | 23063 | 23012 |
| 79 | T * | C * | G | C | A | C | G | G | G | C | A | G |
| 80 | T * | C * | G | C | A | C | G | G | G | C | A | G |
| 81 | T * | C * | G | C | A | C | G | G | G | C | A | G |
| 82 | T | C | G | C * | A * | C * | G | G | G | C | A | G |

*, the base of mutation site was detected with Sanger sequencing, which was undetected using the one-step multiplex HRM with Ct value over 35.
